# Membrane expression of TRAIL receptors DR4, DR5, DcR1 and DcR2 in the normal endometrium, atypical endometrial hyperplasia and endometrioid adenocarcinoma: a tissue microarray study

**DOI:** 10.1007/s00404-013-2840-x

**Published:** 2013-04-13

**Authors:** Leszek Gottwald, Janusz Piekarski, Robert Kubiak, Jarosław Szwalski, Grażyna Pasz-Walczak, Piotr Sęk, Michał Spych, Jacek Suzin, Wiesław Tyliński, Arkadiusz Jeziorski

**Affiliations:** 1 Department of Radiotherapy, Medical University of Lodz, ul Paderewskiego 4, 93-509 Lodz, Poland; 2 Department of Surgical Oncology, Medical University of Lodz, Lodz, Poland; 3 Department of Pathology, Medical University of Lodz, Lodz, Poland; 4 Histopathology Unit, Olympus Consilio Company Ltd, Lodz, Poland; 5 Department of Gynecology and Gynecologic Oncology, Medical University of Lodz, Lodz, Poland

**Keywords:** Tissue microarray, TRAIL receptors, Normal endometrium, Atypical endometrial hyperplasia, Endometrial adenocarcinoma

## Abstract

**Purpose:**

To evaluate the membrane expression of DR4, DR5, DcR1 and DcR2 in the normal endometrium (NE), atypical endometrial hyperplasia (AEH) and endometrioid adenocarcinoma (EAC).

**Methods:**

The study comprised 197 patients: 20 NE, 18 AEH and 159 EAC. Tissue microarrays were constructed. Membrane expression of DR4, DR5, DcR1 and DcR2 was examined and presented as total score (TS).

**Results:**

In EAC, the membrane expression of DR4, DR5 and DcR2 was less common compared to NE (*p* < 0.001; *p* < 0.001; *p* = 0.018) and AEH (*p* < 0.001; *p* < 0.001; *p* = 0.004). In EAC the membrane expression of DcR1 did not differ when compared to NE (*p* = 0.055) and AEH (*p* = 0.173). A strong correlation was found between the type of endometrial tissue (NE/AEH/EAC) and the TS of DR4 (*p* < 0.001), DR5 (*p* < 0.001), DcR1 (*p* = 0.033) and DcR2 (*p* < 0.001). In EAC, the TS of DR4, DR5, DcR1 and DcR2 was not related to grading and staging. In EAC, the membrane expression of DR5, but not DR4, DcR1 and DcR2, was related to better disease-free survival (DFS). The overall survival (OS) was not related to membrane TRAIL receptors expression.

**Conclusions:**

The membrane expression of the receptors for TRAIL DR4, DR5, DcR1 and DcR2 is greater in NE than EAC. The level of membrane staining of the receptors in EAC is not dependent on grading and staging. In EAC patients, membrane expression of DR4, DR5, DcR1 and DcR2 are not independent predictors of survival.

## Introduction

Programmed cell death (apoptosis) is a crucial process in the development and homeostasis of multicellular organisms, and the dysfunction of apoptosis is regarded as an important step in the development of cancer and spreading of metastases [1, 2]. The cell death program consists of three essential types of elements: activators, inhibitors and effectors. The cytokine TNF-related apoptosis-inducing ligand (TRAIL), also known as Apo-2L, is one of the most important extracellular activators of apoptosis. TRAIL is a type II transmembrane protein located on chromosome 8p21–22, that selectively induces apoptosis in tumor cells while leaving normal cells intact [1–5].

The ligand TRAIL binds to five receptors. Two of them, DR4 (TRAIL-R1) and DR5 (TRAIL-R2) are membrane-bound and contain a death domain in their intracellular portion, which is able to transmit an apoptotic signal (“death receptors”). In contrast, the soluble receptor osteoprotegerin is incapable of transmitting an apoptotic signal. Similarly, two other membrane-bound receptors—DcR1 (TRAIL-R3), which lacks the complete intracellular portion and DcR2 (TRAIL-R4), which has a truncated cytoplasmic death domain—do not transmit an apoptotic signal (“decoy receptors”) [6, 7].

The expression of TRAIL and its receptors has been widely described in numerous normal and cancerous tissues [8–13]. Although endometrial cancer is the most common gynecologic malignancy, to date there have been only a few studies attempting to evaluate the expression of TRAIL receptors [14, 15].

The aim of our study is to assess the membrane expression of DR4, DR5, DcR1 and DcR2 in the normal endometrium (NE), atypical endometrial hyperplasia (AEH) and endometrioid adenocarcinoma (EAC) using the tissue microarray method.

## Materials and methods

### Tissue collection

The study examined endometrial tissues from surgery in 197 consecutive patients who had undergone hysterectomy in the Pirogow Memorial Hospital of Lodz between 2000 and 2007. Twenty patients were treated due to uterine fibroids presented with a NE, 18 patients were diagnosed with AEH, and in 159 patients EAC was identified. The distribution of EAC patients by grade was: G1, 59 (37.1 %); G2, 82 (51.6 %); G3, 18 (11.3 %). There were 109 EAC patients at stage I (68.6 %), 24 patients at stage II (15.1 %), and 26 patients at stage III (16.3 %). The patients with EAC were subsequently treated and examined in the Regional Cancer Center, Copernicus Memorial Hospital of Lodz. Disease-free survival (DFS) and overall survival (OS) were analyzed as functions of TRAIL receptor expression. DFS was defined as the period from primary surgery until relapse. OS was defined as the period from primary surgery until the end of the follow-up (60 months) or until the death of the patient. For the study, the approval of the Ethics Committee of the Medical University of Lodz (RNN/82/11/KE) was obtained.

The tissue blocks were fixed in formalin and embedded in paraffin. In all cases hematoxylin–eosin stained slides were available. They were reviewed by a pathologist to confirm the diagnosis of EAC. Additional histological features were recorded: histological grade and FIGO staging (The International Federation of Gynecology and Obstetrics). The patients with EAC were staged according to the FIGO 2009 Staging System [16]. The EAC grading was defined according to the 2003 World Health Organization classification criteria [17]. In each case the NE, the AEH and the EAC was diagnosed in the uterus removed during surgery (not obtained from the curettage).

### Production of tissue microarrays (TMAs)

**Fig. 1:**
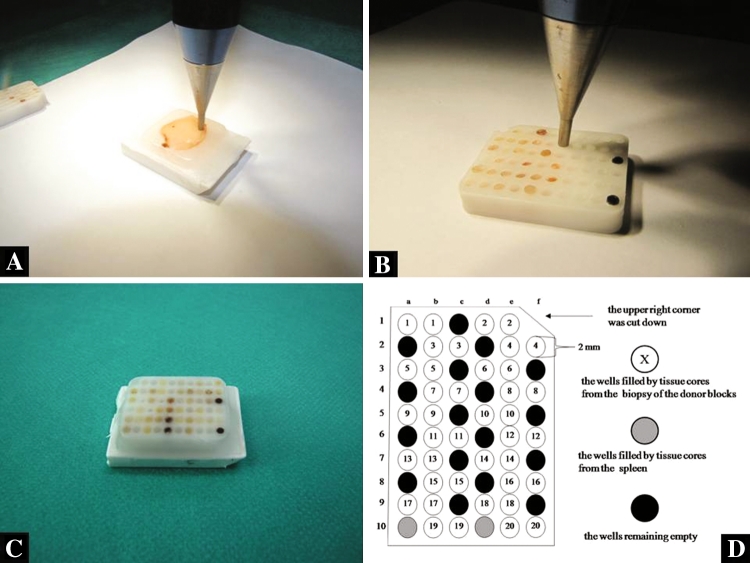
The preparation of the final TMA recipient block: **a** the biopsy technique of the paraffin donor block, **b** the transposition of the tissue core into the wells in the recipient block, **c** the final paraffin recipient block, **d** the sector map of the recipient block: this is a grid that specifies a location within the TMA for each core sample

TMAs were prepared using a manual tissue arraying instrument (Tissue-Tek Quick-Ray Tissue Microarray System; Sakura Finetek USA, Inc. Torrance, CA 90501 USA). Duplicate 2 mm tissue cores were taken from areas representative of NE, AEH and EAC in the donor block, and incorporated into the recipient block (Tissue-Tek Quick-Ray Recipient Block, Sakura Finetek USA, Inc. Torrance, CA 90501, USA) to produce a single recipient block containing 40 cores. To avoid mistakes during identifications of all corners of the TMA recipient block, two wells in the left lower corner of the recipient block were filled by tissue cores from the spleen (Fig. 1a–d). The filled recipient blocks were embedded in paraffin.

### DR4, DR5, DcR1 and DcR2 immunohistochemistry in endometrial tissue

Sections 4 μm thick, cut from TMAs, were used for immunohistochemistry. The sections were deparaffinized in xylene and hydrated in graded ethanol solution. The sections of endometrial tissue were then subjected to pretreatment in order to enhance antigen retrieval. EnVision+ System Horseradish Peroxidase (DakoCytomation, Glostrup, Denmark) and polyclonal antibody to anti-human TRAIL R1/TNFRSF10A—(R&D Systems, Inc. Minneapolis. CA USA), anti-human CD262/TRAILR2 (aa 388–407)—(Acris Antibodies, Inc. San Diego, CA, United States), anti-human TRAIL R3/TNFRSF10C—(R&D Systems, Inc. Minneapolis CA, USA) and anti-human TRAIL R4/TNFRSF10D—(R&D Systems, Inc. Minneapolis, CA, USA) were used for immunohistochemistry. The specificity of the primary antibody had previously been confirmed by the manufacturer. A positive control (positive breast tissue) was used. As a negative control, specimens were immune stained in the absence of primary DR4, DR5, DcR14 and DcR2 antibodies. No immune staining appeared when primary antibodies were not used.

### Immunohistochemical scoring of DR4, DR5, DcR1 and DcR2

**Fig. 2:**
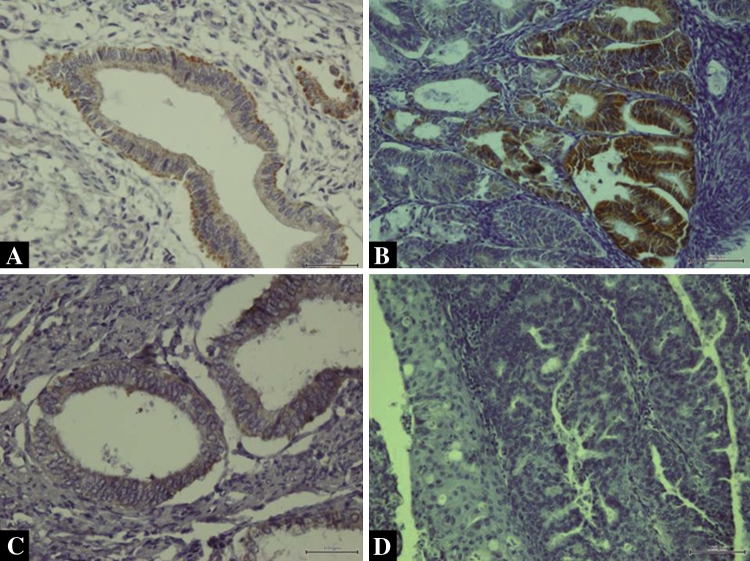
**a** Positive membrane expression of DR4 in NE (×200 magnification), **b** positive membrane expression of DR4 in EAC (×200 magnification), **c** positive membrane expression of DR5 in NE (×200 magnification), **d** lack of DR5 expression in EAC (×200 magnification)

**Fig. 3:**
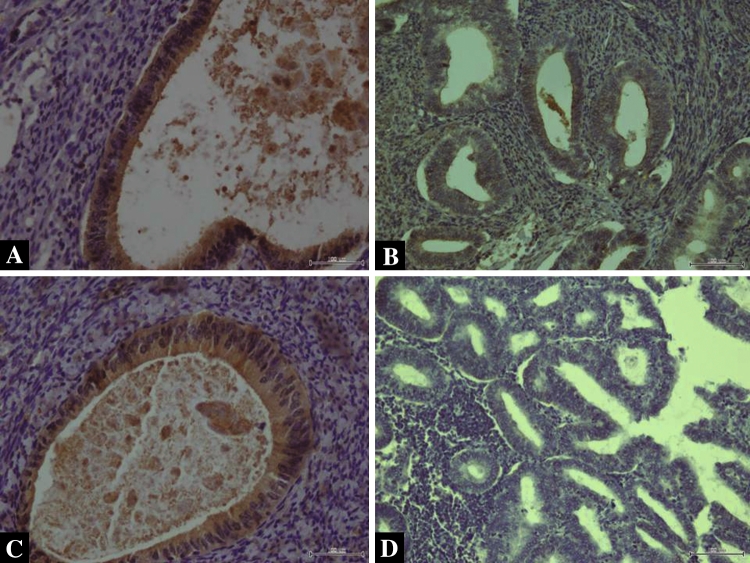
**a** Positive membrane expression of DcR1 in NE (×200 magnification), **b** positive membrane expression of DcR1 in EAC (×200 magnification), **c** positive membrane expression of DcR2 in NE (×200 magnification), **d** lack of DcR2 expression in EAC (×200 magnification)

The specimens were analyzed by independent pathologists with no prior knowledge of the clinical data. The membrane expression of DR4, DR5, DcR1 and DcR2 was assessed in endometrial cells. Both intensity and marker distribution (percentage of the positively stained epithelial cells) were used for the calculation of the scores in the endometrial tissue. The intensity of the staining was scored as follows: 0 = negative, 1 = weak, 2 = moderate, and 3 = strong. Marker distribution was scored as 0 = not present; 1 = 1 %; 2 = 2–9 %; 3 = 10–33 %; 4 = 34–66 %; 5 ≥ 66 %). The final immune staining score was determined by adding the intensity and marker distribution scores in a given case (0 = negative; 2–4 = weak; 5–8 = strong). Examples of endometrial tissue positive and negative for DR4/DR 5 and for DcR1/DcR2 are presented in Figs. 2a–d and 3a–d, respectively.

### Statistical analysis

All results were analyzed using CSS Statistica 9.0 software (Statsoft Inc., Tulsa, OK, USA). The significance of the association between non-parametric data was assessed using the standard Chi-square test and Fisher’s test. Pearson’s *r* correlation coefficient was used to correlate the TS of DR4, DR5, DcR1 and DcR2 expression with the type of endometrial tissue (NE/AEH/EAC), grading and staging of the EAC. Kaplan–Meier survival curves were calculated for patients who were DR4(+)/DR4(−), DR5(+)/DR5(−), DcR1(+)/DcR1(−), DcR2(+)/DcR2(−), as well as for variants of death receptors/decoy receptors. A statistical analysis of survival was performed with the log-rank test and Cox models. A *p* value <0.05 was considered as significant.

## Results

**Fig. 4:**
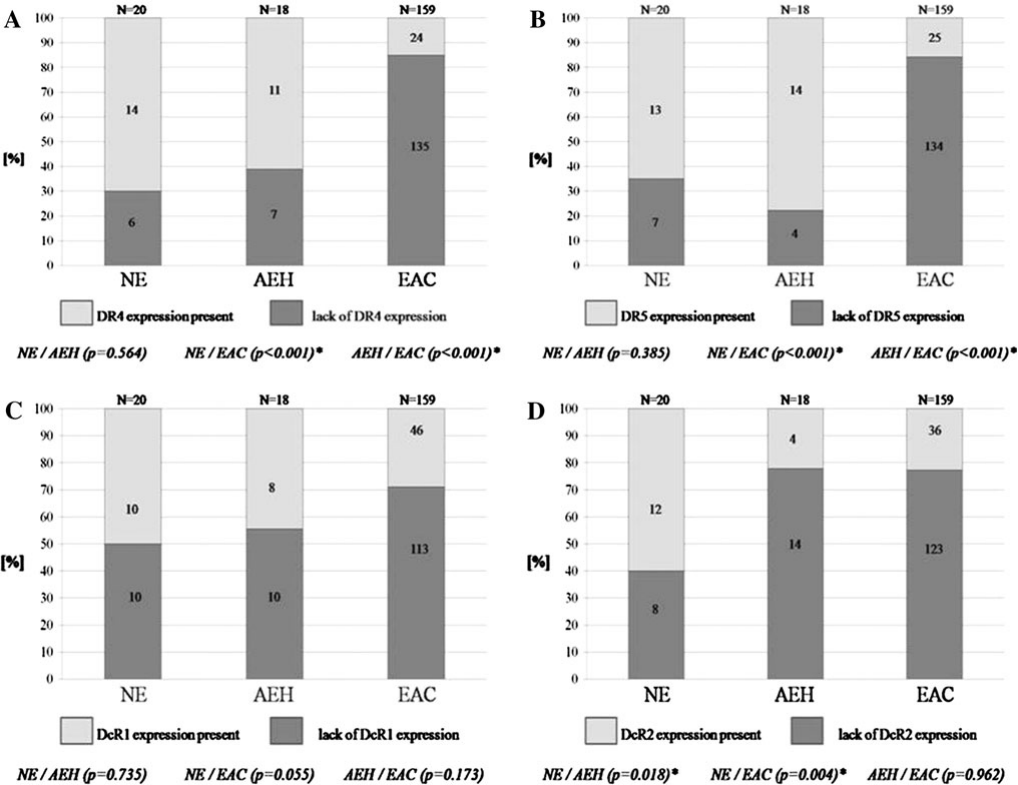
The membrane expression in NE, AEH and EAC of DR4 (**a**), DR5 (**b**), DcR1 (**c**) and DcR2 (**d**)

In EAC the membrane expression of DR4 and DR5 was less common when compared to NE (*p* < 0.001; *p* < 0.001) and AEH (*p* < 0.001; *p* < 0.001). The expression of DR4 and DR5 in NE and AEH was similar (*p* = 0.564; *p* = 0.385) (Fig. 4a, b). A strong correlation between increased TS of DR4 and DR5 in endometrial tissue from NE through AEH to EAC was present (Table 1). In EAC the TS value of DR4 and DR5 was not related to grading (Table 2) and staging (Table 3).

**Table 1:** Cell membrane DR4/DR5/DcR1/DcR2 expression in normal endometrium (NE), atypical endometrial hyperplasia (AEH) and endometrioid adenocarcinoma (EAC)

TRAIL receptors and endometrial tissue	*n*	%	Lack of expression		Weak expression		Strong expression		Pearson’s *r* correlation with TS	*p*
			*n*	%	*n*	%	*n*	%		
DR4 expression										
NE	20	100	6	30.0	5	25.0	9	45.0	−0.469	<0.001*
AEH	18	100	7	38.9	5	27.8	6	33.3		
EAC	159	100	135	84.9	17	10.7	7	4.4		
DR5 expression										
NE	20	100	7	35.0	5	25.0	8	40.0	−0.449	<0.001*
AEH	18	100	4	22.2	4	22.2	10	55.6		
EAC	159	100	134	84.3	12	7.5	13	8.2		
DcR1 expression										
NE	20	100	10	50.0	2	10.0	8	40.0	−0.152	0.033*
AEH	18	100	10	55.5	1	5.5	7	39.0		
EAC	159	100	113	71.1	16	10.0	30	18.9		
DcR2 expression										
NE	20	100	8	40.0	0	0	12	60.0	−0.319	<0.001*
AEH	18	100	14	77.8	0	0	4	22.2		
EAC	159	100	123	77.3	15	9.4	21	13.3		

* Statistical significance

**Table 2:** Cell membrane DR4/DR5/DcR1/DcR2 expression in endometrioid adenocarcinoma (EAC) and grading

TRAIL receptors in EAC and grading	*n*	%	Lack of expression		Weak expression		Strong expression		Pearson’s *r* correlation with TS	*p*
			*n*	%	*n*	%	*n*	%		
DR4 expression										
G1	59	100	49	83.0	4	6.8	6	10.2	−0.059	0.458
G2	82	100	71	86.6	11	13.4	0	0		
G3	18	100	15	83.3	2	11.1	1	5.6		
DR5 expression										
G1	59	100	48	81.3	4	6.8	7	11.9	−0.081	0.313
G2	82	100	70	85.4	6	7.3	6	7.3		
G3	18	100	16	88.8	2	11.2	0	0		
DcR1 expression										
G1	59	100	41	69.5	5	8.5	13	22.0	−0.060	0.455
G2	82	100	58	70.7	9	11.0	15	18.3		
G3	18	100	14	77.6	2	11.2	2	11.2		
DcR2 expression										
G1	59	100	40	67.8	8	13.5	11	18.7	−0.148	0.062
G2	82	100	67	81.7	6	7.3	9	11.0		
G3	18	100	16	88.8	1	5.6	1	5.6

**Table 3:** Cell membrane DR4/DR5/DcR1/DcR2 expression in endometrioid adenocarcinoma (EAC) and FIGO staging

TRAIL receptors in EAC and FIGO staging	*n*	%	Lack of expression		Weak expression		Strong expression		Pearson’s *r* correlation with TS	*p*
			*n*	%	*n*	%	*n*	%		
DR4 expression										
I	109	100	92	84.4	11	10.1	6	5.5	−0.061	0.444
II	24	100	19	79.1	4	16.7	1	4.2		
III	26	100	24	92.3	2	7.7	0	0		
DR5 expression										
I	109	100	92	84.4	8	7.3	9	8.3	−0.006	0.938
II	24	100	19	79.1	3	12.6	2	8.3		
III	26	100	23	88.5	1	3.8	2	7.7		
DcR1 expression										
I	109	100	74	67.9	13	11.9	22	20.2	−0.069	0.386
II	24	100	19	79.1	1	4.2	4	16.7		
III	26	100	20	76.9	2	7.7	4	15.4		
DcR2 expression										
I	109	100	84	77.0	11	10.1	14	12.9	−0.008	0.921
II	24	100	18	75.0	1	4.2	5	20.8		
III	26	100	21	80.1	3	12.2	2	7.7		

**Fig. 5:**
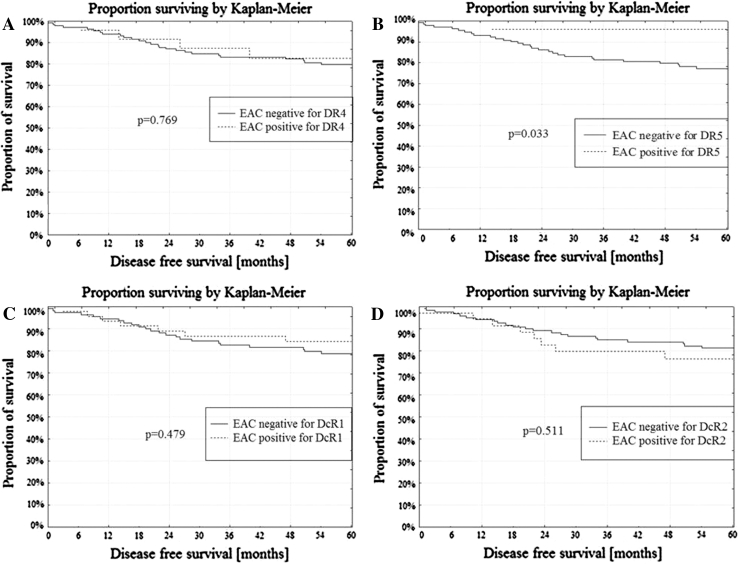
**a** DFS and DR4 membrane expression in EAC, **b** DFS and DR5 membrane expression in EAC, **c** DFS and DcR1 membrane expression in EAC, **d** DFS and DcR2 membrane expression in EAC

**Fig. 6:**
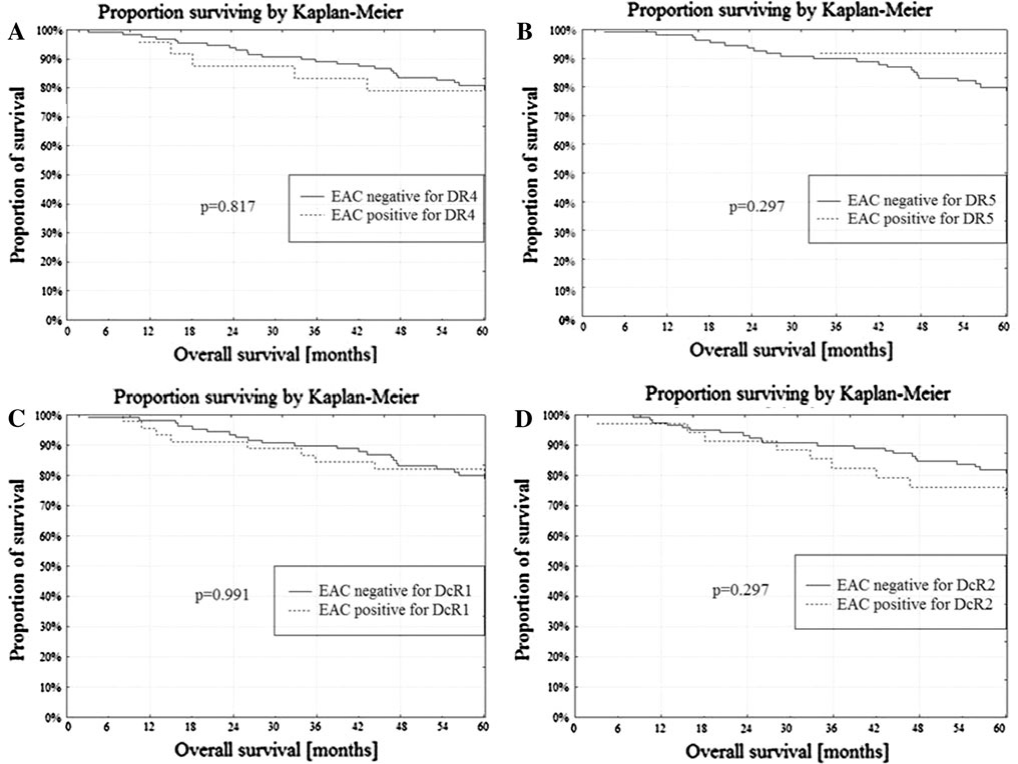
**a** OS and DR4 membrane expression in EAC, **b** OS and DR5 membrane expression in EAC, **c** OS and DcR1 membrane expression in EAC, **d** OS and DcR2 membrane expression in EAC

Membrane expression of both death receptors: DR4 and DR5 was present in 11 NE (55.0 %), 9 AEH (50.1 %) and 10 cases of EAC (6.3 %). Only one receptor DR4 or DR5 expressed 5 NE (25.0 %), 8 AEH (44.4 %) and 29 cases of EAC (18.2 %). Lack of membrane expression of both the DR4 and DR5 receptors was found in 4 NE (20.0 %), 1 AEH (5.5 %) and 120 cases of EAC (75.5 %). The DR4/DR5 receptor status correlated with the type of endometrial tissue from NE through AEH to EAC (*r* = −0.457; *p* < 0.001). In univariate analysis, EAC with positive membrane staining of DR5 presented better DFS when compared to EAC negative for DR5 (*p* = 0.033; Fig. 5b). The membrane expression of DR4 was not related to the DFS (Fig. 5a). The membrane expression of DR4 and DR5 was not related to the OS (Fig. 6a, b).

The expression of DcR1 in NE was lower than in EAC, but not significantly (*p* = 0.055). The presence of DcR1 did not differ between AEH/NE (*p* = 0.735) and AEH/EAC (*p* = 0.173) (Fig. 4c). DcR2 was more frequently expressed in NE than in AEH and EAC (*p* = 0.018; *p* = 0.004). The expression of DcR2 in AEH and in EAC was similar (*p* = 0.962) (Fig. 4d). A correlation was found between increased TS of DcR1 and DcR2 in endometrial tissue from NE to AEH and EAC (Table 1). In EAC the TS value of DcR1 and DcR2 was not related to grading (Table 2) and staging (Table 3).

Membrane expression of both decoy receptors: DcR1 and DcR2 was present in 9 NE (45.00 %), 2 AEH (11.2 %) and 13 cases of EAC (8.2 %), while one receptor, DcR1 or DcR2 was expressed in 4 NE (20.00 %), 8 AEH (44.4 %) and 56 cases of EAC (47.4 %). Lack of membrane expression of both the DcR1 and DcR2 receptors was found in 7 NE (35.0 %), 8 AEH (44.4 %) and 90 cases of EAC (56.6 %). The type of endometrial tissue from NE, AEH and EAC correlated with the DcR1/DcR2 receptor status (*r* = −0.248; *p* < 0.001). The membrane expression of DcR1 and DcR2 was not related to the DFS (Fig. 5c, d) or the OS (Fig. 6c, d).

**Fig. 7:**
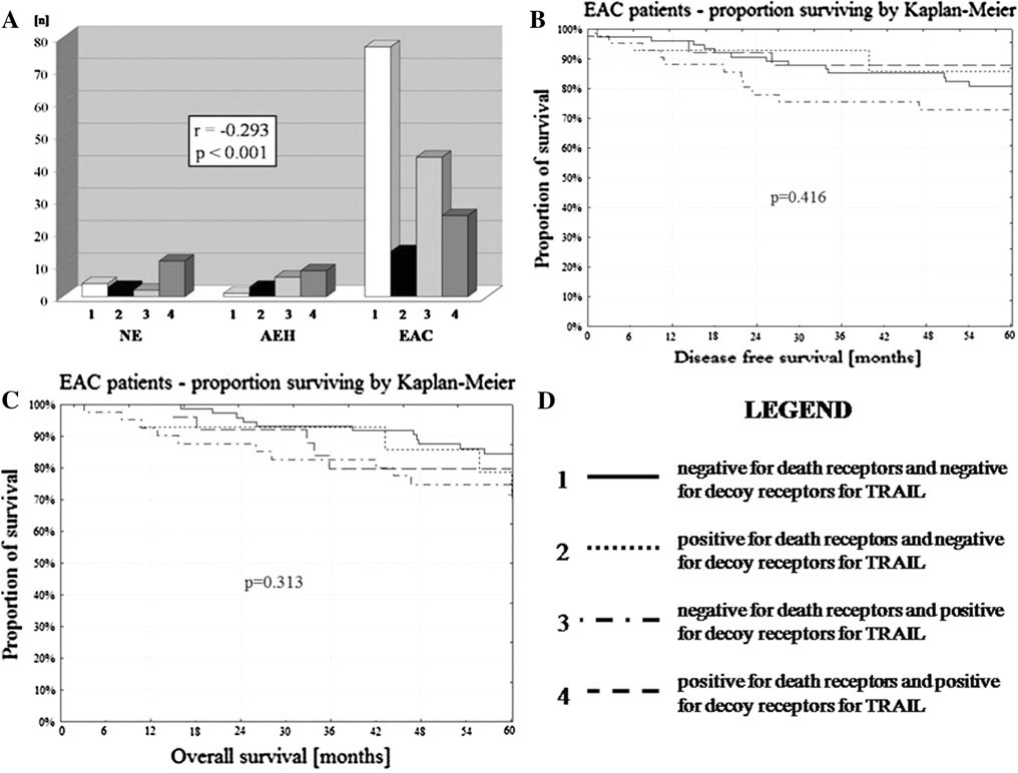
**a** The correlation of the type of endometrial tissue NE/AEH/EAC with variants of TRAIL death/decoy receptor expression. **b** DFS and variants of TRAIL death/decoy receptors. **c** OS and variants of TRAIL death/decoy receptors

The correlation was found between variants of cell membrane death receptor (DR4 + DR5) and decoy receptor (DcR1 + DcR2) expression with the type of endometrial tissue NE/AEH/EAC (*p* < 0.001) (Table 4; Fig. 7a). In the EAC, variants of cell membrane death receptors (DR4 + DR5) and decoy receptors (DcR1 + DcR2) expression were not related to DFS and OS (*p* = 0.416; *p* = 0.313) (Fig. 7b, c).

**Table 4:** The correlation of cell membrane DR4 + DR5 expression (death R) and DcR1 + DcR2 expression (decoy R) with the type of endometrial tissue

DR4 and DR5 expression	NE		AEH		EAC		Pearson’s *r* correlation with tumor type	*p*
	*n*	%	*n*	%	*n*	%		
Death R(−) and decoy R(−)	4	20.0	1	5.6	77	48.4	−0.293	<0.001*
Death R(+) and decoy R(−)	3	15.0	3	16.7	14	8.8		
Death R(−) and decoy R(+)	2	10.0	6	33.3	43	27.0		
Death R(+) and decoy R(+)	11	55.0	8	44.4	25	15.8		
Total	20	100.00	18	100.00	159	100.00		

* Statistical significance

## Discussion

The TMA is a piece of histological equipment designed to efficiently and economically estimate the expression of proteins across large sets of tissue specimens assembled on a single glass microscope slide [18, 19]. The use of TMAs has significant advantages over standard techniques: it allows amplification of limited tissue resources by allowing the production of a large number of small core biopsies, rather than generating a single section saving time, antibodies and costs associated with analyzing multiple specimens at once [18, 20]. Our previous studies confirmed that two 2-mm-sized tissue cores from biopsy of the donor block constructed into the TMAs are sufficient to obtain EAC tissue in above 90 % cases [21].

Since Wiley et al. [1] in 1995 reported the isolation of a new novel member of the TNF ligand family, designated TRAIL, many studies have reported on its significant role in cellular homeostasis. Alterations of TRAIL-induced apoptosis are suspected to be important in the development and progression of cancers, but to date, the mechanism of sensitivity and resistance for TRAIL-induced apoptosis has not been clearly explained [22].

TRAIL receptors show both cell membrane and cytoplasmic staining [9, 11, 12], and in some cases, the receptors can be also expressed in the nucleus [12]. Nowadays it is known that TRAIL and its receptors are present in many normal human tissues, e.g., in peripheral leukocytes, hepatocytes, neurons, renal tubuli contorti, heart myocytes, colonic luminal epithelium and crypt cells, bronchial epithelium and alveolar septa, heart myocytes, germ and Leydig cells [8–10]. Our study also showed membrane staining of DR4, DR5, DcR1 and DcR2 to be present in NE and AEH.

High levels of TRAIL receptor expression were described in the majority of cancer cell lines in vitro, and in many human malignant tumors in vivo [23, 24]. To characterize TRAIL receptors in cancers, cytoplasmic staining is usually analyzed rather than membrane or nuclear staining [9, 12]. The cytoplasmic expression of DR4 and DR5 was found to be strong in primary and metastatic brain tumors, leukemias, malignant melanomas and cancers of the breast, lung, head and neck, esophagus, colorectum, pancreas, renal, urinary bladder, uterine cervix and ovaries [8, 9, 11–13, 25, 26]. Similarly, the cytoplasmic expression of DcR1 and DcR2 was reported, e.g., in primary and metastatic brain tumors [8] and in prostate cancer [27]. In endometrial cancer, DcR1 cytoplasmic expression was evaluated by Tarragona et al. [15]. The authors studied 62 cases of endometrial cancers by tissue microarray, and found DcR1 to be present in 98.1 % of cases [15].

Several recent studies in cancers have reported the occurrence of a correlation between the level of cytoplasmic expression of both death receptors for TRAIL, tumor grading and survival, but the data is generally unclear [12, 25]. For example, Li et al. [25] report a significantly longer postoperative recurrence-free rate in patients with bladder cancer with either high DR4 or DR5 expression, than in those with low expression of both receptors identified during a 10-year follow-up. Koksal et al. [27] report correlation of high cytoplasmic DcR2 expression with high Gleason scores, prostate-specific antigen recurrence and decreased survival. In contrast to this, Koornstra et al. [9] describe a lack of correlation of DR4 and DR5 cytoplasmic expression with grading and staging in cancer of the colon, and Zhuang et al. [28] found DcR1 and DcR2 cytoplasmic expression not to be related to progression of neoplasmic disease. In endometrial cancer patients, Tarragona et al. [15] did not confirm the association of DcR1 expression with histological type of the cancer (EAC vs. non-EAC), its grade and stage, as well.

In contrast to many previous reports, the present study analyzes not cytoplasmic, but also the cell membrane expression of death and decoy receptors for TRAIL in NE, AEH and EAC. It should be noted that although cytoplasmic and nuclear staining of all the receptors was frequently observed during our examination of specimens for DR4, DR5, DcR1 and DcR2, this was not subjected to further analysis. As with other similar studies on breast and esophageal cancers [11, 12], membrane staining of both or one death receptor for TRAIL was found only in 39/159 EAC cases (24.6 %). Interestingly, in contrast to the EAC samples, strong membrane expression of DR4 and/or DR5 was present in 14/20 NE (70.0 %) and 11/18 AEH (61.1 %) cases. Similarly to death receptors for TRAIL, membrane expression of DcR1 and/or DcR2 was present only in 68/159 EAC cases (42.8 %), but in 13/20 NE (65.0 %) and 14/18 AEH (77.7 %) cases. This observation may be a significant step in better characterizing TRAIL-induced apoptosis in normal and malignant cells. Additionally, in our study the intensity of membrane staining of death and decoy receptors for TRAIL in the EAC samples was not found to be correlated with grading, staging and survival. Only membrane expression of DR5 (24 cases) was related to better DFS when compared to patients negative for membrane DR5 (135 cases), which should be verified in further prospective studies in larger populations. The existence of multiple receptors for TRAIL and its different cell distribution suggests that the regulation of signaling by TRAIL cytokine is of a more complex nature [3].

The observation that TRAIL selectively induces cancer cells to undergo apoptosis, while sparing normal cells, has raised great interest in using TRAIL in clinical applications as an anti-tumor weapon [2, 4, 7, 14]. The therapeutic potential of a recombinant soluble human TRAIL has been evaluated in several human tumors, including breast, prostate, glioma and colorectal cancers [29]. Sadarangani et al. [5] describe the positive response of endometrium and endometrial cancer cell lines to TRAIL, and Llobelt et al. [30] demonstrate that endometrial cancer cells, primarily insensitive to TRAIL, can be sensitized to the ligand after administration of chemotherapeutics. These findings are in agreement with other authors using chemotherapeutics in many other forms of cancer, not only gynecologic ones [14, 31, 32]. However, the possibility of applying TRAIL in clinical cancer therapy should be performed with great caution due to the reported possible toxicity of TRAIL to primary human hepatocytes, especially in patients with a diseased liver [29, 33, 34].

In conclusion, our study demonstrates that malignant transformation of the endometrial tissue is related to the reduction of membrane expression of DR4, DR5, DcR1 and DcR2. The level of membrane staining for receptors of TRAIL in the EAC is not dependent on grading and staging of the cancer. Analysis of cell membrane expression of the receptors for TRAIL is not a good predictor of survival in EAC patients. However, the small number of patients used for this study demands further prospective studies in larger populations to confirm these results and to assess its value in clinical practice.

## References

[1] Wiley SR, Schooley K, Smolak PJ, Din WS, Huang CP, Nicholl JK, Sutherland GR, Smith TD, Rauch C, Smith CA (1995). Identification and characterization of a new member of the TNF family that induces apoptosis. Immunity.

[2] Wajant H, Pfizenmaier K, Scheurich P (2002). TNF-related apoptosis inducing ligand (TRAIL) and its receptors in tumor surveillance and cancer therapy. Apoptosis.

[3] Sheridan JP, Marsters SA, Pitti RM, Gurney A, Skubatch M, Baldwin D, Ramakrishnan L, Gray CL, Baker K, Wood WI, Goddard AD, Godowski P, Ashkenazi A (1997). Control of TRAIL-induced apoptosis by a family of signaling and decoy receptors. Science.

[4] Lee YJ, Heurta-Yepez S, Vega M, Baritaki S, Spandidos DA, Bonavida B (2007). The no TRAIL to yes TRAIL in cancer therapy (review). Int J Oncol.

[5] Sadarangani A, Kato S, Espinoza N, Lange S, Llados C, Espinosa M, Villalon M, Lipkowitz S, Cuello M, Owen GL (2007). TRAIL mediates apoptosis in cancerous but not normal primary cultured cells of the human reproductive tract. Apoptosis.

[6] MacFarlane (2003). TRAIL-induced signaling and apoptosis. Toxicol Lett.

[7] Newsom-Davis T, Prieske S, Walczak H (2009). Is TRAIL the holy grail of cancer therapy?. Apoptosis.

[8] Frank S, Köhler U, Schackert G, Schackert H (1999). Expression of TRAIL and its receptors in human brain tumors. Biochem Biophys Res Comm.

[9] Koornstra JJ, Kleibeuker JH, van Geelen C, Rijcken F, Hollema H, de Vries EG, de Jong S (2003). Expression of TRAIL (TNF-related apoptosis-inducing ligand) and its receptors in normal colonic mucosa, adenomas and carcinomas. J Pathol.

[10] Spierings DC, de Vries EG, Vellenga E, van den Heuvel FA, Koornstra JJ, Wesseling J, Hollema H, de Jong S (2004). Tissue distribution of the death ligand TRAIL and its receptors. J Histochem Cytochem.

[11] Younes M, Georgakis GV, Rahmani M, Beer D, Younes A (2006). Functional expression of TRAIL receptors TRAIL-R1 and TRAIL-R2 in esophageal adenocarcinoma. Eur J Cancer.

[12] Ganten TM, Sykora J, Koschny R, Batke E, Aulmann S, Mansmann U, Stremmel W, Sinn HP, Walczak H (2009). Prognostic significance of tumour necrosis factor-related apoptosis-inducing ligand (TRAIL) receptor expression in patients with breast cancer. J Mol Med.

[13] Yoldas B, Ozer C, Ozen O, Canpolat C, Dogan I, Griffith T, San lioglu S, Ozluoglu LN (2011). Clinical significance of TRAIL and TRAIL receptors in patients with head and neck cancer. Head Neck.

[14] Kendrick JE, Estes JM, Straughn JM, Alvarez RD, Buchsbaum DJ (2007). Tumor necrosis factor-related apoptosis-inducing ligand (TRAIL) and its therapeutic potential in breast and gynecologic cancers. Gynecol Oncol.

[15] Tarragona J, Llecha N, Santacana M, Lopez S, Gatius S, Llobet D, Dolcet X, Palomar-Asenjo V, Gonzalez-Tallada FJ, Matias-Guiu X (2010). DcR1 expression in endometrial carcinomas. Virchows Arch.

[16] Pecorelli S (2009). Revised FIGO staging for carcinoma of the vulva, cervix and endometrium. Int J Gynaecol Obstet.

[17] Silverberg S, Kurman R, Nogales F, Mutter G, Kubik-Huch R, Tavassoli FA, Tavassoli FA, Devilee P (2003). Epithelial tumours and related lesions. World health organization classification of tumors. Pathology and genetics of tumours of the breast and female genital organs.

[18] Chen W, Foran DJ (2006). Advances in cancer tissue microarray technology: towards improved understanding and diagnostics. Anal Chim Acta.

[19] Takikita M, Chung JY, Hewitt SM (2007). Tissue microarrays enabling high-throughput molecular pathology. Curr Opin Biotechnol.

[20] Gulmann C, O`Grady A (2003). Tissue microarrays: an overview. Curr Diagn Pathol.

[21] Gottwald L, Sek P, Piekarski J, Pasz-Walczak G, Kubiak R, Szwalski J, Spych M, Suzin J, Tylinski W, Topczewska-Tylinska K, Jeziorski A (2012). Construction of a tissue microarray with 2 mm-size cores in endometrioid endometrial cancer—factors affecting quality of the recipient block. Biotech Histochem.

[22] Merino D, Lalaoui N, Morizot A, Schneider P, Solary E, Micheau O (2006). Differential inhibition of TRAIL-mediated DR5-DISC formation by decoy receptors 1 and 2. Mol Cell Biol.

[23] Davidovich IA, Levenson AS, Levenson (Chernokhvostov) VV (2004). Overexpression of DcR1 and survivin in genetically modified cells with pleiotropic drug resistance. Cancer Lett.

[24] Tomek S, Horak P, Pribill I, Haller G, Rössler M, Zielinski CC, Pils D, Krainer M (2004). Resistance to TRAIL-induced apoptosis in ovarian cancer cell lines in overcome by co-treatment with cytotoxic drugs. Gynecol Oncol.

[25] Li Y, Jin X, Li J, Jin X, Yu J, Sun X, Chu Y, Xu C, Li X, Wang X, Kakehi Y, Wu X (2012). Expression of TRAIL, DR4, and DR5 in bladder cancer: correlation with response to adjuvant therapy and implications of prognosis. Urology.

[26] Ozawa F, Friess H, Kleeff H, Xu ZW, Zimmermann A, Sheikh MS, Buchler MW (2001). Effects and expression of TRAIL and its apoptosis-promoting receptors in human pancreatic cancer. Cancer Lett.

[27] Koksal IT, Sanlioglu AD, Karacay B, Griffith TS, Sanlioglu S (2008). Tumor necrosis factor-related apoptosis inducing ligand-R4 decoy receptor expression is correlated with high Gleason scores, prostate-specific antigen recurrence, and decreased survival in patients with prostate carcinoma. Urol Oncol.

[28] Zhuang L, Lee CS, Scolyer RA, McCarthy SW, Zhang XD, Thompson JF, Screaton G, Hersey P (2006). Progression in melanoma is associated with decreased expression of death receptors for tumor necrosis factor-related apoptosis-inducing ligand. Hum Pathol.

[29] Wu X, Hui KM (2004). Induction of potent TRAIL-mediated tumoricidal activity by hFLEX/Furin/TRAIL recombinant DNA construct. Mol Ther.

[30] Llobet D, Eritja N, Yeramian A, Pallares J, Sorolla A, Domingo M, Santacana M, Gonzalez-Tallada FJ, Matias-Guiu X, Dolcet X (2010). The multikinase inhibitor sorafenib induces apoptosis and sensitizes endometrial cancer cells to TRAIL by different mechanisms. Eur J Cancer.

[31] Moxley KM, Chengedza S, Mangiaracina D (2009). Induction of death receptor ligand-mediated apoptosis in epithelial ovarian carcinoma: the search for sensitizing agents. Gynecol Oncol.

[32] Straughn JM, Oliver PG, Zhou T, Wang W, Alvarez RD, Grizzle WE, Buchsbaum DJ (2006). Anti-tumor activity of TRA-8-anti-death receptor 5 (DR5) monoclonal antibody in combination with chemotherapy and radiation therapy in a cervical cancer model. Gynecol Oncol.

[33] Jo M, Kim TH, Seol DW, Espen JE, Dorko K, Billiar TR, Strom SC (2000). Apoptosis induced in normal human hepatocytes by tumor necrosis factor-related apoptosis-inducing ligand. Nat Med.

[34] Volkmann X, Fischer U, Bahr MJ, Ott M, Lehner F, Macfarlane M, Cohen GM, Manns MP, Schulze-Osthoff K, Bantel H (2007). Increased hepatotoxicity of tumor necrosis factor-related apoptosis-inducing ligand in diseased human liver. Hepatology.

